# Supplementary material for: Content-rich biological network constructed by mining PubMed abstracts
Source: BMC Bioinformatics. 2004 Oct 8;5:147. doi: 10.1186/1471-2105-5-147 (PMC528731; doi:10.1186/1471-2105-5-147)
Supplement: Additional File 2 — The original results of the above study (non-essential files are deleted to keep the file size under the limit set by BMC bioinformatics). [file 1471-2105-5-147-S2.bz2 › chilibotAdditionalFile2/dip05/120ID8668348E464/html/YWHAH_RAF1.html]

 


 **YWHAH** and **RAF1** 
  
Found 3 abstracts in PubMed, retrieved 3.  
 

 What does Google say? 
 PDF only 
| .edu only 

---

- J Biol Chem, 1998   **A novel sphingosine dependent protein kinase SDK1 specifically phosphorylates certain isoforms of 14 3 3 protein.** .
  Protein kinases activated by sphingosine or N,N dimethylsphingosine, but not by other lipids, have been detected and are termed sphingosine dependent protein kinases SDKs .
  These SDKs were previously shown to phosphorylate endogenous 14 3 3 proteins Megidish, T.
  , White, T.
  , Takio, K.
  , Titani, K.
  , Igarashi, Y.
  , and Hakomori, S.
  1995 Biochem.
  Biophys.
  Res.
  Commun.
  21 39 747 .
  We have now partially purified one SDK, termed SDK1, from cytosol of mouse Balb c 3T3 A31 fibroblasts.
  SDK1 is a serine kinase with molecular mass 50 60 kDa that is strongly activated by N, N dimethylsphingosine and sphingosine, but not by ceramide, sphingosine 1 phosphate, or other sphingo , phospho , or glycerolipids tested.
  Its activity is inhibited by the protein kinase C activator phosphatidylserine.
  Activity of SDK1 is clearly distinct from other types of serine kinases tested, including casein kinase II, the alpha and zeta isoforms of protein kinase C, extracellular signal regulated mitogene activated protein kinase 1 Erk 1 , Erk 2, and Raf 1 **[RAF1]**.
  SDK1 specifically phosphorylates certain isoforms of 14 3 3 eta **[YWHAH]**, beta, zeta but not others sigma, tau .
  The phosphorylation site was identified as Ser\* in the sequence Arg Arg Ser Ser\* Trp Arg in 14 3 3 beta.
  The sigma and tau isoforms of 14 3 3 lack serine at this position, potentially explaining their lack of phosphorylation by SDK1.
  Interestingly, the phosphorylation site is located on the dimer interface of 14 3 3.
  Phosphorylation of this site by SDK1 was studied in 14 3 3 mutants.
  Mutation of a lysine residue, located 9 amino acids N terminal to the phosphorylation site, abolished 14 3 3 phosphorylation.
  Furthermore, co immunoprecipitation experiments demonstrate an association between an SDK and 14 3 3 in situ.
  Exogenous N, N dimethylsphingosine stimulates 14 3 3 phosphorylation in Balb c 3T3 fibroblasts, suggesting that SDK1 may phosphorylate 14 3 3 in situ.
  These data support a biological role of SDK1 activation and consequent phosphorylation of specific 14 3 3 isoforms that regulate signal transduction.
  In view of the three dimensional structure of 14 3 3, it is likely that phosphorylation by SDK1 would alter dimerization of 14 3 3, and or induce conformational changes that alter 14 3 3 association with other kinases involved in signal transduction.

  - J Biol Chem, 1997   **Interaction of the ligand activated glucocorticoid receptor with the 14 3 3 eta **[YWHAH]** protein.** .
    The glucocorticoid receptor GR is a ligand activated transcription factor.
    In this study, we used the yeast two hybrid system to isolate cDNAs encoding proteins that interact with the human GR ligand binding domain LBD in a ligand dependent manner.
    One isolated cDNA from a HeLa cell library encoded the COOH terminal portion of the eta isoform of the 14 3 3 protein residues 187 246 .
    Glucocorticoid agonists, triamcinolone acetonide and dexamethasone, induced the GR LBD 14 3 3eta protein fragment interaction, but an antagonist, RU486, did not.
    Glutathione S transferase pull down experiments in vitro showed that full length 14 3 3eta protein also interacted with the activated GR.
    Transient transfection studies using COS 7 cells revealed a stimulatory effect of 14 3 3eta protein on transcriptional activation by the GR.
    The 14 3 3 family members have recently been found to associate with a number of important signaling proteins, such as protein kinase C and Raf 1 **[RAF1]**, as functional modulators.
    Our findings suggest a novel regulatory role of 14 3 3eta protein in GR mediated signaling pathways and also point to a mechanism whereby GR may cross talk with other signal transduction systems.

    - J Biol Chem, 1995   **Identification of the site of interaction of the 14 3 3 protein with phosphorylated tryptophan hydroxylase.** .
      The 14 3 3 protein family plays a role in a wide variety of cell signaling processes including monoamine synthesis, exocytosis, and cell cycle regulation, but the structural requirements for the activity of this protein family are not known.
      We have previously shown that the 14 3 3 protein binds with and activates phosphorylated tryptophan hydroxylase TPH, the rate limiting enzyme in the biosynthesis of neurotransmitter serotonin and proposed that this activity might be mediated through the COOH terminal acidic region of the 14 3 3 molecules.
      In this report we demonstrate, using a series of truncation mutants of the 14 3 3 eta **[YWHAH]** isoform expressed in Escherichia coli, that the COOH terminal region, especially restricted in amino acids 171 213, binds indeed with the phosphorylated TPH.
      This restricted region, which we termed 14 3 3 box I, is one of the structural regions whose sequence is highly conserved beyond species, allowing that the plant 14 3 3 isoform GF14 could also activate rat brain TPH.
      The 14 3 3 box I is the first functional region whose activity has directly been defined in the 14 3 3 sequence and may represent a common structural element whereby 14 3 3 interacts with other target proteins such as Raf 1 **[RAF1]** kinase.
      The result is consistent with the recently published crystal structure of this protein family, which suggests the importance of the negatively charged groove like structure in the ligand binding.
